# The increased prevalence of depression and anxiety in T2DM patients associated with blood glucose fluctuation and sleep quality

**DOI:** 10.1186/s12902-022-01147-8

**Published:** 2022-09-17

**Authors:** Wei Yang, Min Liu, Yuan Tian, Qianwei Zhang, Jiahua Zhang, Qiaoyun Chen, Lixia Suo, Yang Chen

**Affiliations:** 1 Department of Endocrinology, Jiading District Central Hospital Affiliated Shanghai University of Medicine & Health Sciences, Shanghai, China; 2 Department of Hospital Infection Control, The Fifth Affiliated Hospital, Sun Yat-Sen University, Zhuhai, Guangdong China; 3 Department of Neurology, The 904Th Hospital of PLA, Medical School of Anhui Medical University, Wuxi, Jiangsu, China

**Keywords:** Anxiety, Depression, Sleep quality, Blood glucose fluctuation, T2DM

## Abstract

**Background:**

Current evidence demonstrates that blood glucose fluctuation can be associated with depression and anxiety. The association among blood glucose fluctuation, traditional risk factors and emotional disorders in T2DM should be studied and clarified.

**Methods:**

A total of 182 diabetic patients including 81 patients with depression or anxiety and 101 patients without emotional disorder were enrolled into this study. Data were obtained through medical history and questionnaire survey. Data were analyzed using appropriate statistical methods.

**Results:**

The comparison results of basic information between the two groups showed that the differences of the proportion of female were statistically significant (*p* = 0.002).

There was no statistical difference in laboratory examination indexes between the two groups, however, standard deviation of blood glucose (SDBG) and postprandial glucose excursion (PPGE) of the comorbidity group were significantly higher than that of control group (*p* = 0.032 and *p* = 0.037). The results of questionnaire survey showed that there were statistically significant differences in sleep quality, PSQI and dietary habit between the two groups (*p* < 0.001, *p* < 0.001 and *p* < 0.001). Stratified analysis results according to gender showed that the percentage of cognitive disorder, anxiety and depression in female group was significantly higher than that in male group (*p* = 0.001, *p* < 0.001 and *p* < 0.001). Mini-mental state examination (MMSE), self-rating anxiety scale (SAS) and patient health questionnaire (PHQ-9) score in female group were also higher than male group (*p* = 0.001, *p* < 0.001 and *p* < 0.001). Logistic regression analysis results showed that SDBG and sleep quality were associated with emotional disorders in T2DM (*p* = 0.040 and *p* < 0.001) and the OR values of these factors were 7.588 (1.097–52.069) and 4.428 (2.649–7.401).

**Conclusions:**

Blood glucose fluctuation and sleep quality are associated with the increased prevalence of depression and anxiety disorders in T2DM.

## Introduction

Despair, depression, anxiety are the common negative emotions in type 2 diabetes. Some studies indicated that the prevalence of anxiety and cognitive impairment were significantly increased in diabetic patients [1–4]. The proportion of depressive symptoms in diabetic patients is increasing gradually, the rate of depression in diabetes is approximate 20%-30%. More serious consequences occur in diabetes accompanied with emotional disorder patients [5–7]. An investigation including 328 T2DM was conducted by Liu's research group and the results showed that the prevalence of anxiety disorders in the patients with complications was significantly higher compared with diabetic patients without complications (48.76% VS. 24.33%) [8]. It showed that emotional disorders were associated with diabetic complications. Bogner et al. have suggested that the higher risk of death was found in diabetic patients combined with depression and anxiety [9]. Endocrinologists suggested that early identification of emotional disorders and multidisciplinary therapy could improve the prognosis of the disease, reduce the incidence of complications and mortality [10, 11]. Recent studies showed that blood glucose fluctuation was closely related to multiple complications, depression and anxiety in T2DM [12, 13]. Standard Deviation of Blood Glucose (SDBG), postprandial glucose excursion (PPGE) and largest amplitude of glycemic excursions (LAGE) were the common indicators of blood sugar fluctuations, which might be associated with depression and anxiety in diabetic patients.

Therefore, to assess the psychological condition of diabetic patients is beneficial for accurate diagnosis, treatment and blood glucose control. Early identification of poor mental state and potential risk factors are helpful to improve the life quality of diabetic patients.

## Materials and methods

### Study population

A total of 182 T2DM, who were hospitalized in endocrinology department of Jiading District Central Hospital from June 2019 to Apr 2021, became the research subjects of this study. The enrolled patients were distributed into comorbidity group and control group according to the diagnosis of anxiety and depression. After screening, the number of patients was 81 in the comorbidity group and 101 in the control group.

### Inclusion and exclusion criteria

#### Inclusion criteria

- i) T2DM was diagnosed according to the WHO diagnostic criteria in 2013.
- ii) Anxiety and depression must be clearly diagnosed.
- iii) Patients must have sufficient data for the study, including basic information and clinical examination results.

#### Exclusion criteria

- i) Patients with mental disorders who cannot complete questionnaires and assessment scales were excluded.
- ii) Diabetic patients with severe acute complications (serious infection, ketoacidosis, hyperosmolar coma, diabetes foot), hepatic or renal insufficiency, heart deficiency, malignant tumor, malignant anemia, had surgery should be ruled out.
- iii) Patients receiving hormone therapy or antidepressant drug treatment were excluded.

### Research methods and emotional disorders assessment

A questionnaire survey regarding the general information (such as age, gender, body mass index (BMI), smoking status, family history and other medical history materials), behaviors, life style and frequency of food consumption was conducted among the 182 enrolled participants. Meanwhile, the trained physicians assessed the emotional disorders by a variety of scales. The self-rating anxiety scale (SAS) was used to evaluate the state of anxiety. There are 20 items in SAS, the score of each item (range: 1–4) was depending on the severity. The final score was equal to the total score of 20 items multiplied by 1.25 and the standard score was greater than or equal to 50 indicate anxiety. Depressive position was estimated using PHQ-9 scale (0–4: no depression, 5–9: minor depression, 10–14: moderate depression, 15–19: moderately severe major depression, 20–27: severe major depression). Cognitive disorder was recognized by mini mental status examination (MMSE). The total score of the scale was 30 points and the person who score greater than 27 points was thought to be healthy. All patients signed the medical informed consent and agreed to participate in this research. Meanwhile, this study was approved by our local ethics committee.

### Biochemical examination

Venipuncture was used to obtain venous blood and the samples were frozen in -70℃ refrigerator. HbA1c, serum cholesterol, hydrocortisone, thyroid function index, blood calcium, blood phosphorus and other indicators were tested in the two groups. Serum biochemicals were measured by automatic biochemical analyzer (Roche D/P/ISE, Switzerland) and HbA1c was measured by high-performance liquid chromatography (HLC-723g7, Japan). C-peptide and cortisol were tested by the method of chemiluminescent immunoassay (Abbott architect i2000 and AutoLumo A2000Plus, USA).

### Calculation method of blood glucose fluctuation index

The blood glucose levels of pre-prandial and postprandial 2 h blood glucose of three meals and blood glucose before bedtime were measured, then these glucose values were marked as a, b, c, d, e, f, g, respectively. The blood glucose fluctuation indicators including PPGE, LAGE and SDBG were calculated according to the above glucose levels and the calculation formulas are shown below:

$$\overline{\mathbf{x} }=\frac{a+b+c+d+e+f+g}{7}$$

$$\mathbf{P}\mathbf{P}\mathbf{G}\mathbf{E}=\boldsymbol{ }\frac{\left(b-a\right)+\left(d-c\right)+\left(f-e\right)}{3};$$

$$\mathbf{S}\mathbf{D}\mathbf{B}\mathbf{G}= \sqrt{\frac{{(a-\overline{x })}^{2}+{(b-\overline{x })}^{2}+{(c-\overline{x })}^{2}+{(d-\overline{x })}^{2}+{(e-\overline{x })}^{2}+{(f-\overline{x })}^{2}+{(g-\overline{x })}^{2}}{7-1}}$$

### Statistical method

The software STATA version 12.0 (STATA Corp., College Station, Tex) was used to evaluate the collected data. Data consistent with the normal distribution were presented as mean ± standard deviation (Mean ± SD). The numeration data and categorical variables were compared by chi-square analysis or Fisher's exact test. Differences of continuous variables between the two groups were tested by Student’s t-test. Logistic regression analysis was used to identify the associated factors for depression and anxiety disorders in T2DM. The p value less than 0.05 was considered to be statistically significant.

## Result

### General information and clinical parameters

A total of 182 participants were enrolled into this study. The 81 diabetic patients were distributed into comorbidity group according to the diagnosis of anxiety and depression, the rest of the 101 T2DM were into control group. There were no significant differences in age, BMI, waistline, family history and the ratio of other chronic disease between the two groups. The basic information of the study population and analysis results were shown in Table 1. The results showed that the proportion of female in comorbidity group was significantly higher than the ratio of control group (*p* = 0.002). The clinical characteristics of all the participants were shown in Table 2. Compared with control group, the biochemical markers including thyroid function parameters and indicators of liver and kidney function were balanced and had no statistical differences. However, SDBG and PPGE in comorbidity group were higher than those in control group, and the differences were statistically significant (*p* = 0.032 and *p* = 0.037).

**Table 1:** General information of the study population

	Comorbidity group(*n* = 81)	Control group (*n* = 101)	F/χ^2^	*P*
Gender^a^				
Male	34 (41.98%)	66 (65.35%)	9.92	0.002
Female	47 (58.02%)	35 (34.65%)		
Age	56.11 ± 16.20	53.72 ± 13.61	1.17	0.281
BMI	25.30 ± 6.73	25.31 ± 3.69	0.00	0.990
Waistline	88.46 ± 13.98	90.24 ± 9.87	0.96	0.328
Family history of diabetes	31 38.27%)	46 (45.54%)	0.97	0.324
Hypertension history	45 55.56%)	56 (55.45%)	0.00	0.988
Coronary heart disease history	8 (9.88%)	13 (12.87%)	0.40	0.530
Fatty liver	34 (41.98%)	48(47.52%)	0.58	0.448
Diabetic peripheral neuropathy	41 (50.62%)	44 (43.56%)	0.90	0.343
Diabetic retinopathy	29 (35.80%)	27(26.73%)	1.74	0.188
Atherosclerosis or plaque	54 (66.67%)	68 (67.33%)	0.01	0.925

^a^The difference was statistically significant

**Table 2:** Clinical characteristics of the two groups

	Comorbidity group (*n* = 81)	Control group (*n* = 101)	F/χ^2^	*P*
Hba1c	9.62 ± 2.45	9.90 ± 2.58	0.55	0.457
Triglycerides	1.70 ± 0.85	2.07 ± 1.59	3.46	0.065
Total cholesterol	4.44 ± 1.13	4.40 ± 1.23	0.06	0.811
ALT	27.04 ± 28.75	35.83 ± 36.18	3.18	0.076
AST	22.00 ± 17.52	25.13 ± 23.32	-0.59	0.122
Creatinine, μmol/L	65.55 ± 25.37	69.81 ± 25.18	1.26	0.264
25-dihydroxyvitamin-D	14.98 ± 7.06	16.37 ± 6.80	1.78	0.184
Serum calcium	2.37 ± 0.16	2.39 ± 0.13	0.70	0.404
Serum phosphate	1.16 ± 0.21	1.18 ± 0.18	0.38	0.539
UA	318.5 ± 112.65	347.76 ± 126.66	2.63	0.107
FT3	4.20 ± 2.37	4.06 ± 1.56	0.24	0.626
FT4	13.91 ± 3.57	14.26 ± 3.59	0.41	0.524
TSH	1.97 ± 1.46	1.89 ± 1.21	0.15	0.699
TPO-Ab	40.63 ± 152.11	21.57 ± 69.73	1.19	0.277
TG-Ab	20.24 ± 82.29	25.08 ± 79.97	0.15	0.695
Cortisol (8:00 AM)	344.42 ± 98.11	335.37 ± 127.53	0.23	0.636
Cortisol (16:00 PM)	195.59 ± 86.33	183.56 ± 82.97	0.72	0.399
Cortisol (24:00 PM)	126.53 ± 93.73	124.73 ± 97.32	0.01	0.912
C-peptide level	1.47 ± 0.96	1.61 ± 0.91	0.89	0.346
C-peptide level(1 h)	2.73 ± 2.43	3.60 ± 7.13	0.82	0.366
C-peptide level(2 h)	4.11 ± 3.19	5.17 ± 6.62	1.62	0.205
FBG	8.72 ± 2.67	8.41 ± 2.73	0.55	0.460
PBG(1 h)	13.88 ± 4.58	13.39 ± 3.47	0.52	0.474
PBG(2 h)	16.45 ± 5.33	15.93 ± 4.92	0.45	0.506
SDBG^a^	1.29 ± 1.29	0.98 ± 0.34	4.68	0.032
PPGE^a^	2.61 ± 3.11	1.83 ± 1.36	4.43	0.037
LAGE	7.45 ± 8.34	6.23 ± 2.40	1.72	0.192

^a^The difference was statistically significant

### Comparison of sleep status and life behavior

**Fig. 1:**
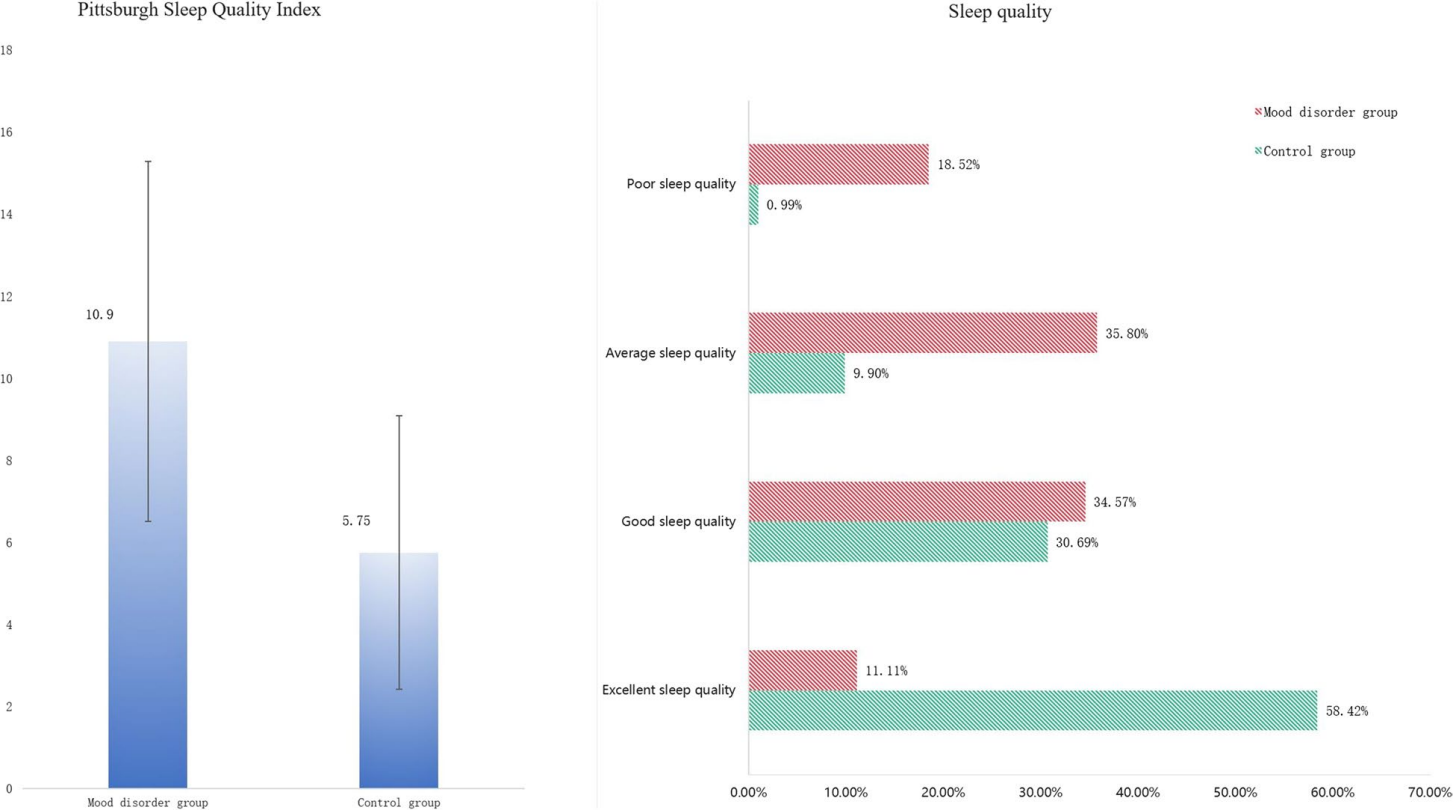
Contrastive analysis of Pittsburgh sleep quality index and sleep quality

In the comorbidity group, people who had the habit of napping account for 66.67%, which was higher than 55.45% in control group, but the difference was not statistically significant (*p* = 0.124). Significant differences of sleep quality and PSQI were existed in the two groups (*p* < 0.001 and *p* < 0.001, Fig. 1). The results of behavioral questionnaires survey (Table 3) showed the habits including alcohol, tea, and smoking habit of the two group had no statistically significant difference (*p* = 0.083, *p* = 0.65 and *p* = 0.095). The difference of dietary structure between comorbidity and control groups was statistically significant (*p* < 0.001).

**Table 3:** The comparison of sleep quality and behavioral style

	Comorbidity group (*n* = 81)	Control group (*n* = 101)	F/χ^2^	*P*
Siesta habit (yes/no)	54/27	56/45	2.37	0.124
Pittsburgh Sleep Quality^a^ Index	10.90 ± 4.39	5.75 ± 3.33	81.03	0.000
Sleep quality^a^				
Excellent	9 (11.11%)	59 (58.42%)	56.91	0.000
Good	28 (34.57%)	31 (30.69%)		
Average	29 (35.80%)	10 (9.90%)		
Poor	15 (18.52%)	1 (0.99%)		
Smoke	24(29.63%)	42 (41.58%)	2.779	0.095
Alcohol	11 (13.58%)	24(23.76%)	3.00	0.083
Tea	31(38.27%)	42 (41.58%)	0.21	0.650
Dietary habit^a^				
Meat-based meal	13(16.05%)	81 (80.20%)	78.86	0.000
Meat pigment mix	54 (66.67%)	10 (9.90%)		
Plant-based diet	14 (17.28%)	10 (9.90%)		

^a^The difference was statistically significant

### Stratification analysis

In this study, there were statistical differences in the gender ratio between the two groups. So the status of anxiety, depression and cognitive disorder in both male and female group were analyzed in this study. The analysis results showed that ratios of cognitive disorder, anxiety and depression in female patients were all significantly higher than that in male diabetes patients (*p* = 0.001, *p* < 0.001 and *p* < 0.001). The differences of MMSE score, SAS score and PHQ9 score were also statistically significant between female T2DM group and male patient group (*p* = 0.001, *p* < 0.001 and *p* < 0.001). The specific analysis results were shown in Table 4. In this research, there was a significant association between gender and depression, thus it was a confounding factor needing to be controlled.

**Table 4:** Anxiety, depression and cognitive disorder status in male and female group

	Male (*n* = 100)	Female (*n* = 82)	F/χ^2^	*P*
Ratio of cognitive disorder^a^	12(12.00%)	27 (32.93%)	11.72	0.001
Ratio of anxiety^a^	1(1.00%)	12 (14.63%)	12.63	0.000
Ratio of depression^a^	32 (32.00%)	50 (60.98%)	15.28	0.000
MMSE score^a^	28.39 ± 2.61	26.78 ± 3.50	12.58	0.001
SAS score^a^	32.92 ± 7.40	38.11 ± 9.83	16.48	0.000
PHQ-9 score^a^	2.80 ± 3.35	4.59 ± 3.79	11.35	0.000

^a^The difference was statistically significant

### Analysis of the influence factors associated with anxiety and depression in T2DM

Logistic regression analysis was used to identify the influence factors for anxiety and depression disorders in T2DM. According to the comparative analysis results between the two groups, it was found that sex ratio, the blood glucose fluctuation index, sleeping status and dietary habit were statistically significant. Therefore, sex ratio and other traditional risk factors such as smoking and alcohol consumption were considered as potential confounders. After controlling the confounders, the results showed that SDBG and sleep quality were associated with depression and anxiety disorders in T2DM (*p* = 0.040 and *p* < 0.001) and the OR values of these factors were 7.588 (1.097–52.069) and 4.428 (2.649–7.401), respectively. The male–female ratio, age, BMI, smoke, alcohol and dietary habit were not associated with depression and anxiety in T2DM (*p* = 0.801, *p* = 0.393, *p* = 0.337, *p* = 0.652, *p* = 0.489 and *p* = 0.828, separately). PPGE, LAGE and MBG had no effects on depression and anxiety in T2DM (*p* = 0.437, *p* = 0.180 and *p* = 0.836). The analysis results were shown in Table 5.

**Table 5:** Analysis of factors related to anxiety and depression in T2DM

	OR	95% CI	*P*
Male–female ratio	0.860	(0.266, 2.778)	0.801
Age	0.987	(0.958, 1.017)	0.393
BMI	1.040	(0.960, 1.126)	0.337
Smoke	0.769	(0.245, 2.408)	0.652
Alcohol	0.651	(0.193, 2.193)	0.489
Sleep quality^a^	4.428	(2.649, 7.401)	0.000
Dietary habit	1.076	(0.555, 2.084)	0.828
SDBG^a^	7.558	(1.097, 52.069)	0.040
PPGE	0.880	(0.637, 1.215)	0.437
LAGE	0.829	(0.631, 1.090)	0.180
MBG	0.984	(0.848, 1.142)	0.836

^a^The differences were statistically significant

## Discussion

In the current study we found that SDBG was significantly associated with depression and anxiety in T2DM (*p* = 0.032) and blood sugar that fluctuated widely was associated with depression and anxiety (OR = 7.558, *p* = 0.040). The T2DM patients having poor self-regulating ability might lead to a wide range of blood glucose fluctuations, multiple complications were emerging including common complications, psychological and emotional diseases [14–16]. The reason of SDBG fluctuation associated with the prevalence of depression and anxiety might be that long-term blood glucose disorder and increased complications could cause the proportion of anxiety and depression increasing.

Our results comported with several prior studies that good sleep quality, health of dietary patterns and regular behaviors were considered as the advantage factors, which could improve the depressive symptoms [17–19]. Logistic analysis results showed that sleep disorder was the risk factor for depression and anxiety in T2DM patients (OR = 4.428, 95%CI: 2.649–7.401, *p* < 0.001). The traditional risk factors such as smoking and alcohol, were not statistically associated with depression and anxiety in this study (*p* = 0.652 and *p* = 0.489). The cause might be that gender imbalance between the two groups and insufficient sample size.

According to Hussain's systematic review analysis, the prevalence of depression was 26.67% ~ 29% in diabetes [20]. However, the prevalence of emotional distress in our study was 45.05%, the increased prevalence of anxiety and depression might be probably associated with diagnostic mistakes, delayed therapy. Peyrot team's findings confirmed that gender was the independent risk factors for emotional disorders in diabetic patients [21]. Our results were consistent with those research findings, the female patients were more likely to suffer from anxiety, depression and cognitive disorder (*p* = 0.001, *p* < 0.001 and *p* < 0.001). As we all know that menopause can lead to endocrine disorders and emotional fluctuation in women, this might account for the difference in gender.

This research was a rigorous retrospective study and focused on the correlation of blood glucose fluctuation, sleep quality and the prevalence of depression and anxiety in T2DM. However, it should be noted that there were some limitations in this research. The research data were collected from the electronic medical record and questionnaires, it was impossible to eliminate information bias, selection bias and confounding bias completely. We can only minimize the effects of these biases by collecting data objectively and reasonable statistical analysis. Large-sample and multicenter studies were needed to clarify the causal relationship between blood glucose fluctuation and emotional disorders in T2DM.

## Conclusion

To conclude, this retrospective analysis indicated that blood glucose fluctuation and sleep quality were associated with the increased prevalence of depression and anxiety in T2DM. It is known that early identification of poor mental state and potential risk factors are helpful to improve the life quality of diabetic patients.

## Data Availability

The datasets generated and/or analysed during the current study are not publicly available due to personal data protection legislation but are available from the corresponding author on reasonable request.

## Abbreviations

T2DM: Type 2 diabetes mellitus
BMI: Body Mass Index
HbA1c: Glycosylated hemoglobin
UA: Uric Acid
FBG: Fasting Blood Glucose
PBG: Postprandial blood glucose
TSH: Thyroid Stimulating Hormone
FT3: Free Triiodothyronine
FT4: Free Thyroxine
ALT: Alanine transaminase
AST: Aspartate transaminase
TG-Ab: Thyroglobulin Antibody
TPO-Ab: Thyroid Peroxidase Antibody
LAGE: Largest amplitude of glycemic excursions
PPGE: Postprandial glucose excursion
SDBG: Standard Deviation of Blood Glucose
PSQI: Pittsburgh sleep quality index
MMSE: Mini-mental state examination
SAS: Self-Rating Anxiety Scale
PHQ-9: Patient health questionnaire
